# Role of fine-needle aspiration cytology and core biopsy in the preoperative diagnosis of screen-detected breast carcinoma

**DOI:** 10.1038/sj.bjc.6603211

**Published:** 2006-06-06

**Authors:** B Lieske, D Ravichandran, D Wright

**Affiliations:** 1 Bedfordshire and Hertfordshire Breast Screening Unit and Luton & Dunstable Breast Unit, Luton & Dunstable Hospital, Lewsey Road, Luton, Bedfordshire LU4 0DZ, UK

**Keywords:** fine-needle aspiration cytology, core biopsy, breast screening, breast carcinoma

## Abstract

Core biopsy (CB) has now largely replaced fine-needle aspiration cytology (FNAC) in the preoperative assessment of breast cancer in the UK. We studied the contribution of FNAC and CB in the preoperative diagnosis of screen-detected breast carcinoma. Data were prospectively collected on 150 840 women who underwent breast screening over a 4-year period from 1999 to 2003. Data on women who had both FNAC and CB taken from the same lesion preoperatively and in whom surgical excision of the lesion subsequently confirmed malignancy was analysed. In 763 cancers, FNAC was inadequate (C1) in 8% and benign (C2) in 10%. Most of these cases presented with microcalcification (25% were C1 or C2). Core biopsy was not representative (B1) or benign (B2) in 7%. The absolute and complete sensitivities were 65 and 82% for FNAC and 80 and 93% for CB in the diagnosis of cancer. Core biopsy was abnormal (B3 or above) in 86% of the cancers missed by FNAC and FNAC was abnormal (C3 or above) in 65% of those missed by CB. Core biopsy is better than FNAC at preoperative diagnosis of screen-detected breast cancer as it missed fewer cancers. However, combining FNAC resulted in a better preoperative diagnosis rate.

The National Health Service Breast Screening Programme (NHSBSP) was introduced in the United Kingdom in 1987, following publication of the findings of an expert committee the previous year. At present, all women aged 50–70 years are offered two-view screening mammography every 3 years. Patients with radiologically suspicious lesions are recalled for further assessment that may include clinical examination, special mammographic views, ultrasound and collection of tissue for pathological assessment by fine-needle aspiration cytology (FNAC), core biopsy (CB), or more recently, by wide bore vacuum biopsy.

In the early stages of NHSBSP, FNAC was the test used in the assessment. Core biopsy was introduced in the assessment process in late 1990s. The experience with this technique has improved considerably and CB is now considered to be the standard. The preoperative diagnosis rate of screen-detected carcinoma has been improving with increasing use of CB. Correspondingly, the use of FNAC is in decline and a number of screening units in the UK have abandoned it completely (Britton *et al*, 1997).

At the Bedfordshire and Hertfordshire Breast Screening Unit, CB was introduced in the assessment of screen-detected breast lesions in 1997, but we continued to perform FNAC in addition in the majority of patients with suspicious lesions. The aim of this study was to assess the performance of FNAC and CB in the preoperative diagnosis of screen-detected breast carcinoma.

## MATERIALS AND METHODS

Data were prospectively collected on all women who attended the Bedfordshire and Hertfordshire Breast Screening Unit, England. We reviewed a 4-year period from April 1999 to March 2003. This period was chosen to ensure that operators had gained sufficient expertise in the use of CB, which was introduced in 1997. We selected, for detailed analysis, the women who had both FNAC and CB performed at the preoperative diagnostic assessment and had malignancy confirmed by subsequent surgery. In patients who have had more than one assessment (if they were recalled for further biopsies, etc), only the initial assessment was considered.

Fine-needle aspiration cytology and CB were performed under image guidance (either ultrasound or stereotaxis), or clinically by a breast radiologist or breast physician. Fine-needle aspiration cytology was performed first using a 21 G needle attached to a 10 ml syringe, and CB was performed using an automated device (14 G). When FNAC was performed under stereotaxis, five needle passes were routinely undertaken, and under ultrasound or clinical guidance 1–2 passes were performed. When CB was guided by stereotaxis, five passes were routinely undertaken, and the specimens were checked for calcification by radiography if appropriate. If no calcification was obtained as many passes as reasonably possible were made until calcium was retrieved. Two to three passes were undertaken when CB was clinically or sonographically guided. Local anaesthetic (LA) was not used for FNAC, except when FNAC was performed under stereotaxis, where multiple passes were undertaken. When LA was used, FNAC was performed using a different needle to that which was used to administer the LA. Local anaesthetic was always used for CB. Immediate evaluation of the FNAC was not performed routinely. The outcomes of FNAC and CB were reported using the standard NHSBSP criteria (Table 1). Sensitivity of FNAC and CB were calculated in two ways (Britton, 1999): absolute sensitivity included only C5 or B5 results and complete sensitivity included C3, C4 and C5 for FNAC and B3, B4 and B5 for CB. We were unable to calculate the specificity of tests, as radiologically nonsuspicious lesions producing benign FNAC and CB are not surgically excised in our practice. The miss rate for CB was defined as the proportion of all breast cancers (invasive and ductal carcinoma *in situ* (DCIS)) with a diagnosis of only benign findings (B1 or B2) on CB.

## RESULTS

During the study period, 150 840 women underwent breast screening and 5285 were recalled for further assessment of a screen-detected lesion in the breast, an overall recall rate of 3.5%. Among these, 2092 had both FNAC and CB performed and 869 of these patients proceeded to surgery where histology confirmed malignancy (DCIS or invasive) in 763 cases. Another 902 patients had either FNA (*n*=803) or CB (*n*=99) but not both, and 72 patients in this group were also diagnosed with cancer (total number of cancers 835), which gives an overall cancer detection rate of 5.53/1000 in this group. Those 763 patients who had both FNAC and CB, in whom malignancy was subsequently confirmed by surgery, constitute the study population. Six hundred and twenty-five patients had breast-conserving surgery and 138 had mastectomy.

The final histology consists of invasive ductal carcinoma (IDC) (*n*=408, 53%), invasive lobular carcinoma (ILC) (*n*=93, 12%), mixed ductal and lobular carcinoma (*n*=33, 4%) or tubular carcinoma (TC) (*n*=32, 4%) with or without DCIS. One hundred and seventy-six patients (23%) had DCIS only. The remaining 21 patients had invasive carcinomas of other types.

### Mammographic presentation

The mammographic lesions were classified as microcalcification in 231 cases and a soft tissue lesion (mass, mass associated with microcalcification, asymmetrical density or stromal deformity) in 532 cases.

### Mode (clinical, stereotactic or US) by which FNAC and CB were performed

The data on the mode by which FNAC and CB were performed are presented in Table 2. In 73% of patients (*n*=555), both tests were performed using the same mode. Relatively more FNACs were performed clinically without image guidance compared with CB (*n*=208 and 86, respectively).

### Outcome of FNAC and CB (Table 3)

Absolute sensitivity, which considers only the definitely malignant (C5 or B5) results, was 80% for CB and 65% for FNAC. Complete sensitivity, which considers all abnormal results (B3 and above and C3 and above), was 93 and 82%, respectively. When both tests are combined, the absolute sensitivity was 87% and complete sensitivity was 98%. Complete sensitivity of FNAC varied with the final histology: it was 89% for IDC, 73% for ILC, 81% for mixed ductal and lobular carcinoma, 72% for TC and 73% for DCIS. Corresponding figures for CB were 92, 98, 94, 86 and 94%, respectively. Overall, the complete sensitivity of CB was higher than that of FNAC regardless of whether the tumour was DCIS or invasive, mammographic presentation (microcalcification or soft tissue lesion) or the mode (clinical, US or stereotaxis) of biopsy (Table 4). Core biopsy suggested (B3 or above) 86% of the cancers missed by FNAC and FNAC was abnormal (C3 or above) in 65% of those missed by CB. When only the 555 patients who had both tests performed under the same modality (clinical, US or stereotaxis) were considered, the absolute and complete sensitivities were 62 and 78% for FNAC and 80 and 93% for CB, respectively. The corresponding figures for the combination were 82 and 97%.

### Nondiagnostic/benign FNAC and CB

Core biopsy was not representative of the lesion (B1) in 36 patients (5%) and was benign (B2) in 17 patients (2%). Fine-needle aspiration cytology was inadequate (C1) in 61 patients (8%) and was benign (C2) in 74 patients (10%). When both tests were combined, the number of cancers producing inadequate/nonrepresentative samples in both tests (any combination of C1, C2 and B1, B2) reduced to 19 (2.5%). These were diagnosed by a subsequent assessment (*n*=8) or open biopsy. The miss rate for CB was 6.9%.

Table 5 summarises C1, C2, B1 and B2 results in relation to mammographic presentation, mode of biopsy and final histology. Fine-needle aspiration cytology was inadequate (C1) or benign (C2) mostly in lesions presenting as microcalcifications (12.5% were C1 and 12.5% were C2), with stereotactic approach (13 and 15%) and with lesions proved to be DCIS (13.6 and 14%). Fine-needle aspiration cytology was benign (C2) in 19% of ILCs.

## DISCUSSION

Accurate preoperative diagnosis of carcinoma is necessary in screen-detected lesions so that patients may be counselled appropriately and a majority could have a single therapeutic operation. Fine-needle aspiration cytology is a very useful test, relatively rapid and inexpensive, less invasive owing to finer needle size and is easier/safer in certain lesions, such as very small lesions, lesions just under the skin or very close to the chest wall compared with CB. In addition, FNAC maintains tactile sensitivity, allows multidirectional passes allowing a broader sampling of the lesion and immediate reporting where necessary. It is used extensively in our screening centre as well as elsewhere. However, it is less reliable at differentiating invasive cancer from DCIS, may be limited in some cases in the assessment of tumour grade and prognostic/predictive markers such as hormone receptors and Her-2/neu, has a relatively high inadequate rate and is more time consuming for pathologists. Cores can be radiographed to reveal microcalcification to confirm accurate targeting of the lesion. These factors have made CB now the test of choice in screen-detected breast carcinoma in NHSBSP in the UK. Among 13 290 cancers detected by the UK NHSBSP in the year 2003/2004, 93% were diagnosed preoperatively and only 8% were diagnosed by cytology alone, the rest by CB only or a combination of CB and FNAC (ABS at BASO, 2005). In this study, our aim was to explore the value of performing both FNAC and CB in the preoperative assessment of screen-detected breast carcinoma. An initial indeterminate FNAC result (C3 and C4) or CB result (B3 and B4) in our institution would lead to further assessment; either further sampling of the lesion by core biopsies, vacuum-guided biopsies or surgery or, occasionally, with less suspicious lesions, an early recall of patients for further mammograms and assessment in 6–12 months time. Thus, we consider complete sensitivity (C3 and above and B3 and above) as truly representative of the diagnostic ability of the test, as a cancer is likely to be picked up eventually.

The better preoperative diagnosis rate of CB in our study was mainly owing to better diagnosis of DCIS, which mainly presented with mammographic microcalification that often required stereotactic approach. It is known that the sensitivity of FNAC is the lowest when performed stereotactically (Britton, 1999), especially in the assessment of microcalcifications (Pisano *et al*, 1998), and our findings in this large study confirm this observation. Cores performed for microcalcification were radiographed to confirm the presence of calcium, which obviously improved the adequacy of samples contributing to the improved diagnosis of DCIS by CB. However, even with malignancy presenting as microcalcifications, the complete sensitivity increased from 94% for CB only to 98% for the combination of FNAC and CB.

To compare tests fairly, both FNAC and CB should be taken from the same lesion that is later surgically excised for definitive histology. Both tests should be performed in the same sitting, ideally using the same guidance (clinical, US or stereotactic), and the operator should be skilled in both techniques. Fine-needle aspiration cytology, especially, is more operator-dependent than CB. An adequately trained and experienced cytopathologist is necessary to reduce the number of nondiagnostic FNACs, and the interpretation of cytology is relatively more dependent on the expertise of the cytopathologist than CB is on the quality of histopathologist. Despite the large amount of literature on FNAC and CB in breast diseases, only a few relatively small studies have been reported, where both FNAC and CB were taken from the same lesion in the same sitting and compared. Early studies compared FNAC with a single ‘Tru-Cut’ needle biopsy in palpable cancers. Results were variable and the performance of CB was often suboptimal, with nondiagnostic biopsies in up to a third of patients in some studies (Elston *et al*, 1978; Shabot *et al*, 1982; Dixon *et al*, 1986; Cheung *et al*, 1987; Khanna *et al*, 1991). More recent studies in palpable lesions have usually used automated CB devices with relatively larger needle sizes and usually found CB to be more sensitive. When both tests were performed clinically in palpable lesions, the sensitivity of FNAC varied from 90 to 98% and that of CB from 90 to 100%, with one study showing better sensitivity for FNAC than CB (Ballo and Sneige, 1996; Agarwal *et al*, 2003; Dennison *et al*, 2003). When both tests were performed under ultrasound guidance, some found the sensitivity of the FNAC to be equal to that of CB (Hatada *et al*, 2000; Westenend *et al*, 2001), whereas others showed CB to be better (Chuo and Corder, 2003). In 112 breast cancers from our symptomatic unit, FNAC did not provide useful additional information owing to CB correctly diagnosing nearly all cancers leaving little room for FNAC to improve upon the preoperative diagnosis rate (Pilgrim and Ravichandran, 2005).

Fewer comparison studies of similar nature have been reported with screen-detected breast cancers. In an early study of 76 cancers, stereotactic FNAC (20 G, 2–3 passes) showed better complete sensitivity and less absolute sensitivity than stereotactic CB (20 G, 2–3 passes), but all sensitivities were generally low (32–72%) (Dowlatshahi *et al*, 1991). In another study of single-pass stereotactic FNAC and CB (18 G) in 65 nonpalpable breast cancers (Lifrange *et al*, 1997), FNAC was inadequate in 22% and benign in 34%. The corresponding figures for CB were 3 and 38%. It is now known that a minimum of five, often more, cores are necessary, especially with microcalcification, to reduce the number of inadequate specimens and false-negatives (Liberman *et al*, 1994; Romanelli and Smith, 1999; Michell, 2000). In a study of 81 carcinomas presenting as microcalcification, complete sensitivity of stereotactic FNAC (up to three passes) was 65% compared with 97.5% for stereotactic CB (14 G, 5–10 passes) (Newman *et al*, 2001). There is a risk that when FNAC is performed as an adjunct to CB, it may be relegated to a second place and is not as satisfactorily performed or evaluated as it would have been if it were the only diagnostic test performed, resulting in poor sensitivity and specificity with high inadequate rates (Ibrahim *et al*, 2001). It has been reported that introducing CB in a screening unit that was previously running a very successful FNAC-based service may result in a reduction in the performance of FNAC (Newman *et al*, 2001). Relatively more FNACs were performed clinically without image guidance in our study compared with CB. This was in part owing to FNAC being used to assess whether the lesion was truly palpable in a situation where a radiologically visible cancer is associated with a vaguely palpable mass at the site. For example, a lesion visible on ultrasonography with a corresponding vaguely palpable abnormality may be sampled by CB under US guidance and by FNAC under clinical guidance. If both confirm malignancy, it may be inferred that the radiological and palpable lesions are the same. However, more FNAC being performed clinically did not appear to have affected the performance of FNAC adversely in our study, as the sensitivity of FNAC was the highest when performed clinically and the inadequate rate lowest. These results, however, probably reflect more of the nature and size of the lesion being sampled rather than the mode of sampling.

Our study confirms an advantage of combining FNAC and CB for screening-detected breast cancers. The complete sensitivity increases from 93% for CB only to 98% when both tests are combined. The increase is similar for both invasive cancers and DCIS. This additional benefit by FNAC is probably owing to its ability to sample a larger area by multidirectional passes of the needle and the maintenance of tactile sensitivity. Adding FNAC to CB results in only minimal additional trauma to the patient, so the combined approach is unlikely to result in extra complications and it would help to maintain the breast cytology expertise locally. However, whether this modest increase in diagnostic sensitivity by additional FNAC is cost effective is difficult to comment, as we have not performed a formal cost-effective analysis. Fine-needle aspiration cytology is inexpensive in terms of disposables (of the order of £1–2, 1.46–2.92€), but it is time consuming for pathologists and this is probably where the real ‘cost’ of FNAC lies. Core biopsy costs approximately £20 (29€) per biopsy in disposables alone. We hoped that it might be possible to identify a subgroup of lesions, which would benefit most from having both tests performed but did not find one. It would, however, be reasonable to undertake CB only in lesions that are clinically or radiologically suspicious/malignant, as these lesions would not be overlooked if the CB were negative.

## Figures and Tables

**Table 1:** Reporting categories for FNAC and for CB

	**Cytology reporting**		**Core biopsy reporting**
C1	Unsatisfactory	B1	Unsatisfactory/normal tissue only
C2	Benign	B2	Benign
C3	Atypia probably benign	B3	Benign, but of uncertain malignant potential
C4	Suspicious of malignancy	B4	Suspicious of malignancy
C5	Malignant	B5	Malignant
			B5a Noninvasive cancer
			B5b Invasive cancer
			B5c Cancer of nonassessable invasiveness

CB=core biopsy; FNAC=fine-needle aspiration cytology.

**Table 2:** Mode of FNAC and CB

	**CB**				
**FNAC**	**Stereo**	**US**	**Clinical**	**Not recorded**	**Total**
Stereo	270	12	1		283
US	59	205	5	1	270
Clinical	28	96	80	4	208
Not recorded	2				2
					
Total	359	313	86	5	763

CB=core biopsy; FNAC=fine-needle aspiration cytology.

**Table 3:** Cytology (C) and core biopsy (B) results of 763 cases in which malignancy was confirmed at surgical excision

	**B1**	**B2**	**B3**	**B4**	**B5**	**Total**
C1	5	2	4	6	44	61
C2	5	7	14	12	36	74
C3	0	2	8	1	17	28
C4	7	3	7	18	68	103
C5	19	3	2	23	449	496
						
Total	36	17	35	60	614	762^a^

a Data on one case incomplete.

**Table 4:** Complete sensitivity (%) of FNAC and CB with regard to histology, mammographic presentation and mode of biopsy (*n*=763)

	**Histology**			**Mammographic presentation**		**Mode of Biopsy**		
	**All Cancers (invasive and DCIS)**	**Invasive**	**DCIS only**	**Microcacification**	**Soft tissue lesion**	**Clinical**	**US**	**Stereo**
FNAC	83 (80)	85 (84)	74 (71)	75 (73)	85 (85)	91 (95)	86 (86)	71 (72)
CB	93 (93)	93 (92)	94 (93)	94 (94)	93 (92)	95 (95)	94 (93)	95 (92)
Combined FNAC and CB	98 (97)	97 (96)	98 (98)	97 (97)	98 (97)	NC (99)	NC (98)	NC (96)

CB=core biopsy; DCIS=ductal carcinoma *in situ*; FNAC=fine-needle aspiration cytology; NC=not calculable.

Numbers within parentheses are those concerning 555 patients who had both tests performed under the same guidance (clinical, US or stereotaxis).

**Table 5:** Percentages of inadequate (C1), nondiagnostic (B1) and benign (C2 and B2) FNAC and CB in relation to mammographic presentation, mode of biopsy and final histology

	**C1**	**B1**	**C2**	**B2**
*Mammographic presentation*				
Microcalcification	12.5	2.6	12.5	3.5
Soft tissue lesion	6	5.6	8.5	1.7
				
*Mode of biopsy*				
Clinical	3.8	3.5	4.8	1.2
US	5.9	5.8	7.8	0.6
Stereotaxis	13.1	3.9	15.2	3.9
				
Final histology				
Invasive ductal	5.6	5.6	5.1	1.7
Invasive lobular	7.5	3.2	19.4	0
Other invasive	7.0	3.5	12.8	4.7
DCIS	13.6	3.4	13.6	3.4

CB=core biopsy; DCIS=ductal carcinoma *in situ*; FNAC=fine-needle aspiration cytology.
